# Association of Racial Residential Segregation and Other Social Determinants of Health with HIV Late Presentation

**DOI:** 10.1007/s10461-024-04535-4

**Published:** 2024-10-17

**Authors:** Fanghui Shi, Jiajia Zhang, Shujie Chen, Xueying Yang, Zhenlong Li, Sharon Weissman, Bankole Olatosi, Xiaoming Li

**Affiliations:** 1 Department of Health Promotion, Education, and Behavior, Arnold School of Public Health, South Carolina SmartState Center for Healthcare Quality, University of South Carolina, 915 Greene Street, Columbia, SC 29208 USA; 2 Department of Health Promotion, Education and Behavior, Arnold School of Public Health, University of South Carolina, Columbia, SC 29208 USA; 3 Department of Epidemiology and Biostatistics, Arnold School of Public Health, University of South Carolina, Columbia, SC 29208 USA; 4 Department of Geography, College of Earth and Mineral Sciences, The Pennsylvania State University, University Park, PA 16802 USA; 5 Department of Internal Medicine, School of Medicine, University of South Carolina, Columbia, SC 29208 USA; 6 Department of Health Services Policy and Management, Arnold School of Public Health, University of South Carolina, Columbia, SC 29208 USA

**Keywords:** Racial Residential Segregation, HIV, Late Presentation, Social Determinants of Health

## Abstract

Understanding social determinants of HIV late presentation with advanced disease (LPWA) beyond individual-level factors could help decrease LPWA and improve population-level HIV outcomes. This study aimed to examine county-level social determinants of health (SDOH) with HIV late presentation. We aggregated datasets for analysis by linking statewide HIV diagnosis data from the South Carolina (SC) Enhanced HIV/AIDS Reporting System and multiple social contextual datasets (e.g., the American Community Survey). All adult (18 years and older) people with HIV diagnosed from 2014 to 2019 in SC were included. Linear mixed models with forward selection were employed to explore the association of county-level SDOH with the county-level three-year moving average percentage of LPWA and average delay time from HIV infection to diagnosis. Around 30% of new HIV diagnoses were LPWA in SC, and the mean delay time for people with LPWA was approximately 13 years. Counties with more racial residential segregation had longer average delay time (Adjusted beta = 5.079, 95% CI: 0.268 ~ 9.889). Regarding other SDOH, the increased percentage of LPWA was associated with fewer Ryan White centers per 100,000 population (Adjusted beta = -0.006, 95% CI: -0.011~-0.001) and higher percentages of the population with less than a high school education (Adjusted beta = 0.008, 95% CI: 0 ~ 0.015). Reducing county-level disparities in LPWA requires multifaceted interventions addressing multiple dimensions of SDOH. Targeted interventions are needed for counties with more Black residential segregation, fewer Ryan White centers, and higher percentages of less than high school education.

**Supplementary Information:**

The online version contains supplementary material available at 10.1007/s10461-024-04535-4.

## Introduction

HIV late presentation with advanced disease (LPWA) is defined as having a record of CD4 count < 200 cells/µL or having an AIDS diagnosis within three months of the initial HIV diagnosis. Being diagnosed with HIV at such a late stage in the HIV disease trajectory has significant impacts on both people with HIV (PWH) and the HIV epidemic. Late diagnosis and late entry into care decrease the life expectancy of PWH and increase the risk of onward HIV transmission in the community [1]. Evidence from immune recovery research suggests that LPWA results in less favorable outcomes, even with efficacious therapies [2]. To fully realize the benefits of available care and treatment, it’s critical to shorten the time lag between HIV infection and diagnosis, especially in resource-limited areas with high HIV disease burden, such as South Carolina (SC) [2]. According to the Centers for Disease Control and Prevention (CDC) HIV/AIDS surveillance data, SC ranked 11th among US states with an AIDS case rate of 8.6 per 100,000 population [3]. Additionally, according to the latest national data from AIDSVu, around 25.8% of new HIV diagnoses in SC were LPWA in 2021, which ranks 12th among the 50 states and is much higher than the national average level of 21.1% [4, 5]. 

The structural socioeconomic and cultural context of HIV should be considered when allocating resources for designing, implementing, and evaluating prevention or intervention programs in different areas. Understanding and addressing social determinants of LPWA beyond individual-level behavioral and biomedical factors could help decrease LPWA and improve population-level HIV outcomes [6]. SC, like many Southern US states, ranks high for uninsured population, low educational attainment, and poverty. These factors affect individuals’ access to prevention and healthcare care, which may lead to a higher risk of LPWA [7–9]. Some social determinants of health (SDOH), such as limited transportation options, poor road infrastructure, concentrated poverty, and high healthcare costs, are frequently reported in HIV service utilization studies, including studies about barriers to HIV testing [2, 10]. However, these barriers are not explicitly reported in HIV late diagnosis studies and need further examination [11, 12]. 

Racial residential segregation is one prominent manifestation of structural racism in the US and has been a key mechanism by which racial/ethnic disparities in health have been created [13]. As one important structural characteristic, racial residential segregation demonstrated a negative impact on varied HIV care continuum outcomes. A systematic review of neighborhood factors impacting HIV care continuum participation in the US found that racial residential segregation was associated with lower CD4 counts, late HIV diagnosis, non-linkage to care, and a lower likelihood of retention in care [14]. For example, Shi F and colleagues found that counties with high racial/ethnic residential segregation were more likely to have low viral suppression rates and compromise the goal of the HIV treatment cascade [15]. Black/African American individuals are more likely to be segregated than other racial/ethnic groups, and communities with high Black concentration are more likely to have limited access to important resources [13]. Even though high HIV testing rates are found among African Americans, HIV late presentation remains a problem that is disproportionately borne by this racial/ethnic group [12]. For instance, a census tract-level study using HIV surveillance data from 2009 to 2013 in New York City found that communities with high Black racial concentration had a higher relative risk of late HIV diagnosis independent of socioeconomic deprivation and income inequality [16]. However, evidence regarding the association between racial residential segregation and LPWA is limited in rural areas such as SC. Further studies are needed due to great heterogeneities in sociocultural characteristics between urban and rural areas.

By linking multiple physical and social contextual data (e.g., American Community Survey [ACS]) and statewide HIV diagnosis data, this study aims to (1) describe the spatiotemporal variations of the county-level three-year moving average percentage of LPWA in SC from 2014 to 2019 and (2) explore the association of racial residential segregation and some other SDOH with LPWA.

## Methods

### Population and Data Sources

The current study was conducted at the county level, encompassing all 46 counties in SC from 2014 to 2019. All adult (≥ 18 years old) PWH diagnosed from January 2014 to December 2019 in SC were included in the current study. We retrieved their information about HIV/AIDS diagnosis date and CD4 test results from the SC Enhanced HIV/AIDS Reporting System (eHARS), which contains confirmed HIV/AIDS cases from all laboratories, hospitals, and health care providers in SC [7]. County-level SDOH were retrieved from multiple sources of physical and social contextual data, including ACS, County Health Rankings (CHR), Area Health Resource Files (AHRF), and the US Department of Health and Human Services (DHHS). The Federal Information Processing Standards (FIPS) code, a unique identifier of each county, was used to link different datasets by county.

### Outcomes

Individual-level LPWA was defined based on whether a person had a CD4 test result < 200 cells/µL or an AIDS diagnosis within the first three months of initial HIV diagnosis. To illustrate the geographic disparities in HIV late diagnosis, county-level 3-year moving average (2014–2016, 2015–2017, 2016–2018, and 2017–2019) percentages of LPWA were calculated as the number of people considered as LPWA divided by the total number of new HIV diagnosis during the three years at each county.

Another outcome we were interested in was the county-level 3-year moving average (2014–2016, 2015–2017, 2016–2018, and 2017–2019) delay time from HIV infection to diagnosis. For individuals with LPWA, their delay time was calculated based on the CD4 depletion model commonly used to estimate the duration from HIV infection to the date of the CD4 test based on the first CD4 value after HIV diagnosis [17, 18]. The detailed information of the model and calculation is described in detail elsewhere [17]. The mean delay time of all individuals with LPWA at each county during three consecutive calendar years was calculated as the county-level 3-year moving average delay time from HIV infection to diagnosis. A total of 184 observations were collected across 46 counties in SC for both outcomes, with each county measured four times across years.

### Racial Residential Segregation

We used five measurements of racial residential segregation to capture different dimensions of residential segregation, including the Black/White dissimilarity index, the White/non-White dissimilarity index, the isolation index, the percentage of Black/African Americans, and the percentage of Hispanics [19]. We extracted the Black/White dissimilarity index and White/non-White dissimilarity index directly from CHR to measure the residential evenness, which refers to the differential distribution of two racial groups among area units [20]. Higher values of dissimilarity indices indicate greater residential segregation between two racial groups. We calculated the Black isolation index to measure the residential exposure, which refers to the degree of potential contact between minority and majority group members within area units [19]. The calculation is:

$$\sum {\:_{{\text{i}} = 1}^{\text{n}}} \left[ \begin{gathered}\left( {\frac{{{\text{Total}}\:{\text{number}}\:{\text{of}}\:{\text{Black}}\:{\text{residents}}\:{\text{in}}\:{\text{census}}\:{\text{tract}}\:}}{{{\text{Total}}\:{\text{number}}\:{\text{of}}\:{\text{Black}}\:{\text{residents}}\:{\text{in}}\:{\text{the}}\:{\text{county}}}}} \right) \hfill \\\left( {\frac{{{\text{Total}}\:{\text{number}}\:{\text{of}}\:{\text{Black}}\:{\text{residents}}\:{\text{in}}\:{\text{census}}\:{\text{tract}}}}{{{\text{Total}}\:{\text{population}}\:{\text{in}}\:{\text{the}}\:{\text{census}}\:{\text{tract}}}}} \right) \hfill \\ \end{gathered} \right]$$

where n is the number of census tracts, and i is the i^th^ census tract in the county [21]. The isolation index ranges from 0 to 1, with larger values indicating greater residential segregation [21]. We also retrieved the percentage of the population reporting Black/African American alone and the percentage of the population reporting Hispanic ethnicity alone from ACS as proxy measures of residential segregation to indicate racial concentration in a county [22]. 

### Other County-Level SDOH

Other county-level factors were selected based on the SDOH framework [23]. They were categorized into four key areas, including social & community context (e.g., population density, the percentage of households with same-sex unmarried partners, and the percentage of housing units that were mobile homes, economic stability (e.g., Poverty%, unemployed%, and Gini index of income inequality), education access (e.g., percentage of population with less than high school education), and healthcare access & quality (e.g., mental health providers rate, the shortage of primary care physicians, the shortage of mental health providers, uninsured%, and the number of Ryan White centers) [23]. The definition and data source of each county-level variable are presented in supplemental Table 1.

### Statistical Analysis

First, choropleth maps were created in ArcGIS to illustrate the spatiotemporal variations of the three-year moving average percentage of LPWA and delay time from HIV infection to diagnosis across four public health regions in SC (e.g., Upstate, Midlands, Pee Dee and Lowcountry) from 2014 to 2019. Second, three quantiles (e.g., 25th percentile, median, and 75th percentile) and interquartile range (IQR) were used to describe all county-level factors and outcomes by year. The Kruskal-Wallis test was used to examine county-level variable variations. Third, two linear mixed models with forward selection were employed to explore the association of racial residential segregation and some other county-level SDOH with the 3-year moving average percentage of LPWA and 3-year moving average delay time, respectively. Longitudinal data is analyzed in this study, and GLMM allows us to account for both fixed and random effects, making it suitable for clustered data where observations within groups (e.g., counties) are not independent. Unique county FIPS code was set as a random effect in our models, and the intraclass correlation coefficient (ICC) was calculated to distinguish the variance attributable to within-county differences from that due to between-county differences. Given a large set of potential covariates in the analyses, the forward selection was chosen to identify the most relevant predictors and balance model simplicity and explanatory power. A p-value of less than 0.2 was used as the criteria for adding variables to the final model during forward selection. We assumed the within-county correlation structure in the model is the Gaussian spatial correlation structure, depending on their distance. SAS software version 9.4 (SAS Institute, Inc., Cary, NC) was used to conduct all statistical analyses. The study protocol was approved by relevant SC state agencies and the institutional review board from the University of South Carolina.

## Results

### Descriptive Statistics

**Fig. 1:**
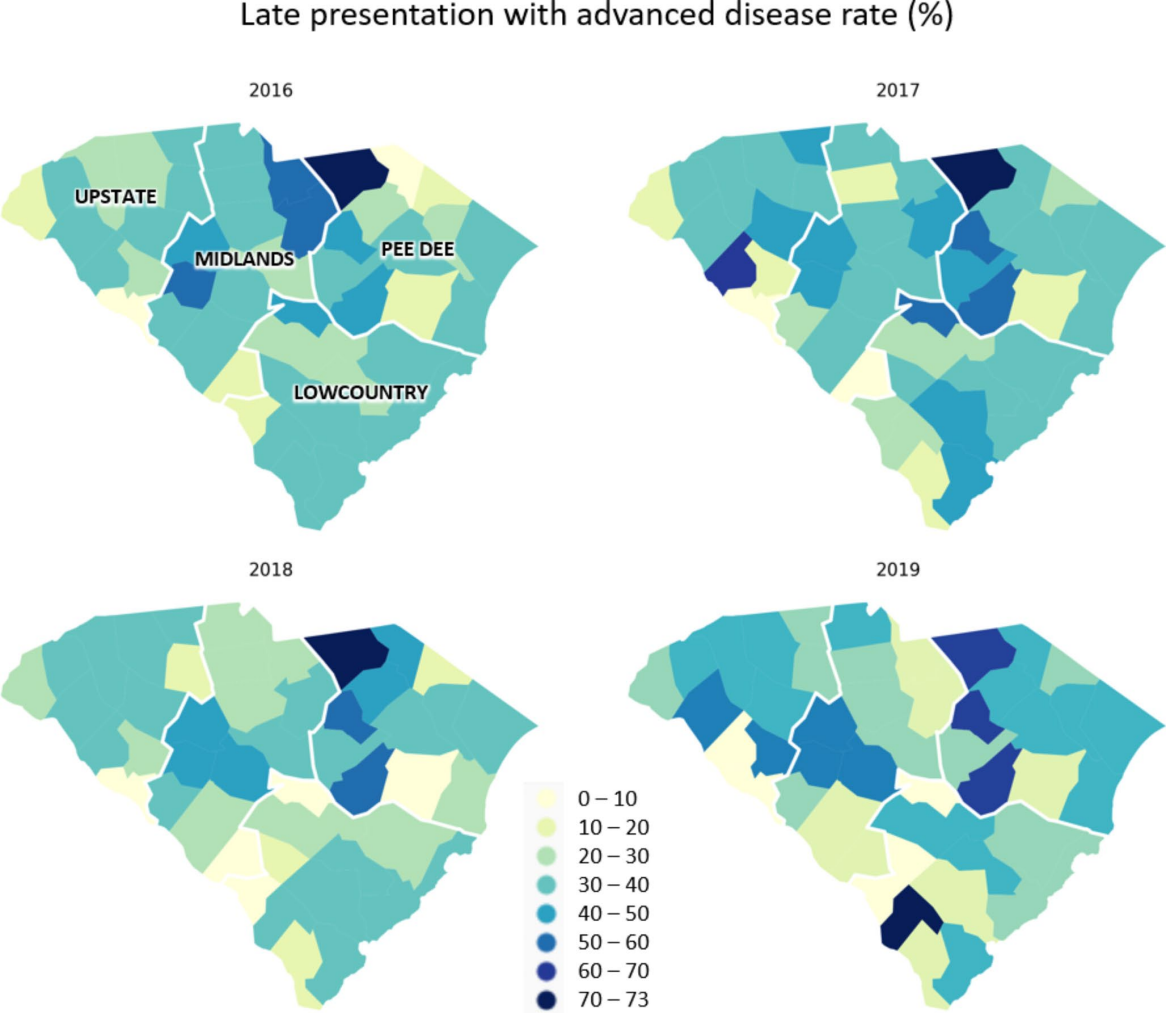
County-level three-year moving average percentage of late presentation with advanced disease among people with HIV in South Carolina

**Fig. 2:**
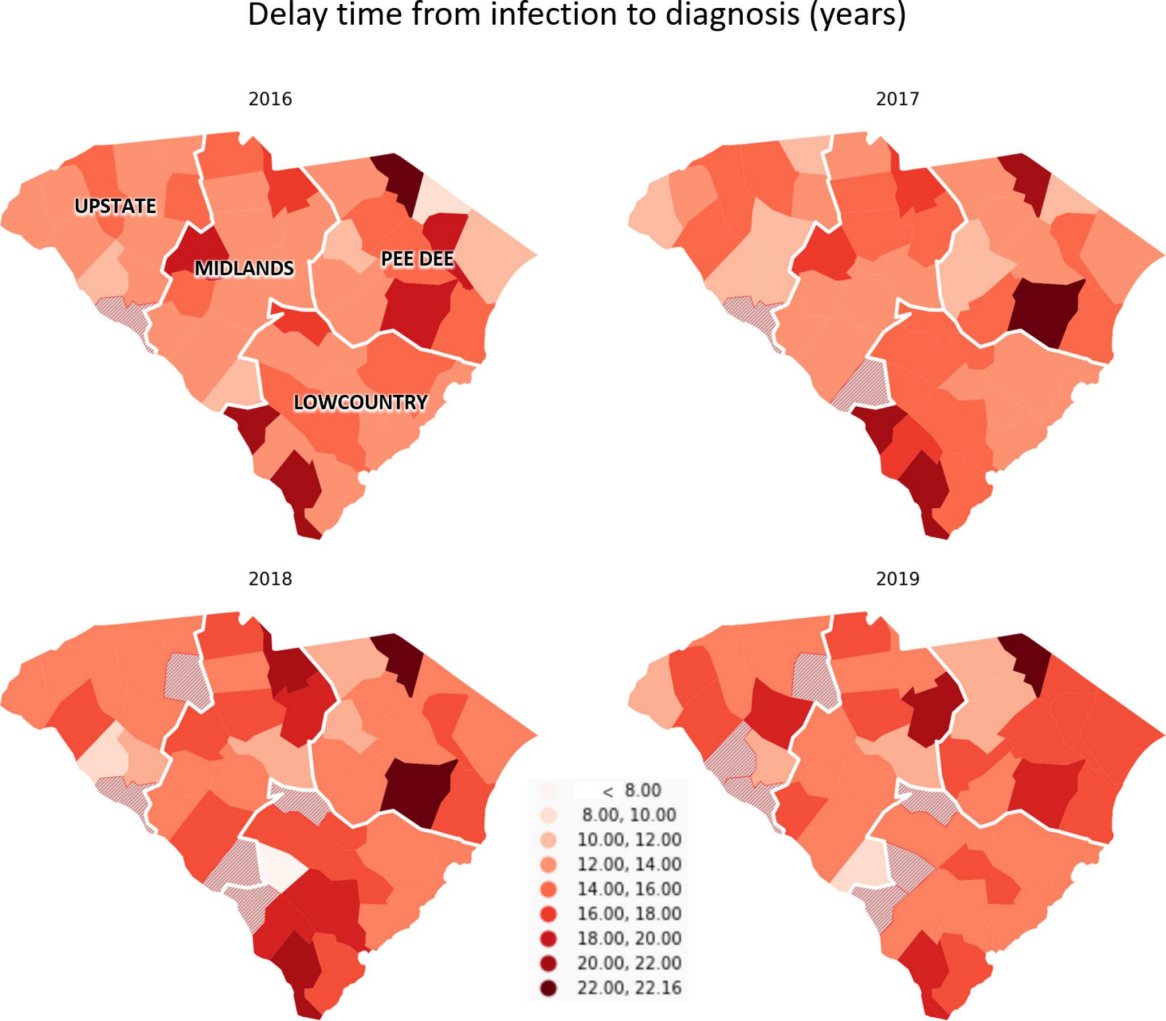
County-level three-year moving average delay time (in years) from HIV infection to diagnosis among people with HIV in South Carolina

Across 46 counties in SC, counties with a percentage of LPWA higher than 60% and an average delay time longer than 22 years were concentrated more in the Pee Dee region. (Figures 1 and 2) From 2016 to 2019, the median percentage of LPWA decreased from 34 to 30%, and the median delay time decreased from 13.78 years to 13.60 years. However, these changes were insignificant (p-values were 0.2075 and 0.9109 for percentage of LPWA and delay time, respectively). Across counties, large variations existed for the percentage of Black persons, with the median value being 32% and IQR larger than 20%. Regarding the variation of county-level variables over the years, significant decreases were observed in the percentage of labor force unemployment and the percentage of population uninsured (p-value < 0.0001). In 2016, the median percentage of Hispanics was 3.6% (IQR: 2.28-5.4%), which increased to 3.93% (IQR: 3.4-5.61%) in 2018. For the isolation index, the median in 2016 was 0.45 (IQR: 0.37–0.59), and it remained relatively stable over the years, with the median being 0.45 (IQR: 0.36–0.6). The Black/White dissimilarity index and the White/Non-White dissimilarity followed a similar trend, with a stable median value of around 30 over the years. (Table 1)

**Table 1:** Descriptive characteristics of county-level percentages of late presentation with advanced disease, delay time, and social determinants of health

Variables	25th percentile	Median	75th percentile	IQR^2^	*p*-value^3^
**Outcomes**					
Percentage of LPWA^1^ (%)					0.2075
2016	28	34	38	10	
2017	29	36	41	12	
2018	27	33	38	11	
2019	20	30	38	18	
Delay time (years)					0.9109
2016	12.99	13.78	14.69	1.7	
2017	12.58	13.94	15.21	2.63	
2018	12.87	13.71	15.14	2.27	
2019	12.75	13.6	14.95	2.2	
**Racial residential segregation**					
Black %					0.9988
2016	24.33	32.08	46.76	22.43	
2018	23.84	32.2	47.02	23.18	
Hispanic %					0.8506
2016	2.28	3.6	5.4	3.12	
2018	2.4	3.93	5.61	3.21	
Isolation index					0.9993
2016	0.37	0.45	0.59	0.22	
2018	0.36	0.45	0.6	0.24	
Black/White dissimilarity index					0.9989
2016	24	30	38	14	
2018	26	30	39	13	
White/Non-White dissimilarity index					0.9982
2016	23	29.5	34	11	
2018	25	29	35	10	
**Social & community context**					
Population density					0.9999
2016	44.41	79.16	180.39	135.98	
2018	44.82	78.08	189.44	144.62	
Same-sex unmarried partner %					0.764
2016	0.1	0.21	0.33	0.23	
2018	0.06	0.2	0.3	0.24	
Mobile homes %					0.9996
2016	18.17	24.25	31.77	13.6	
2018	17.32	24.69	31.82	14.5	
**Economic stability**					
Poverty %					0.5371
2016	15.2	18.25	22.3	7.1	
2018	14.5	18.45	23.5	9	
Unemployed %					< 0.0001
2016	8.02	9.46	10.81	2.79	
2018	5.96	7.42	9.07	3.11	
Median income					0.1068
2016	34,319	40,313	46,328	12,009	
2018	36,276	42,514	49,392	13,116	
Gini index					0.6709
2016	0.45	0.46	0.48	0.03	
2018	0.45	0.47	0.49	0.04	
**Education access**					
Less than high school %					0.1851
2016	14.52	17.69	20.73	6.21	
2018	13.35	16.92	19.21	5.86	
**Health care access & quality**					
Mental health care providers per 100,000					0.2963
2016	15	36	73	58	
2018	12	20.5	57	45	
Shortage of primary care physicians					1
2016	1	2	2	1	
2018	1	2	2	1	
Shortage of mental healthcare providers				.	0.5587
2016	1	1	2	1	
2018	1	1	2	1	
Uninsured %					< 0.0001
2016	12	13	15	3	
2018	10	11	12	2	
Ryan White centers per 100,000 population					1
2016	0.34	1.61	4.56	4.22	
2018	0.33	1.55	4.63	4.3	

Notes: ^1^LPWA (late presentation with advanced disease);

^2^IQR (Interquartile Range)

^3^ p-value comes from Kruskal-Wallis test;

Descriptive values for year 2017 and 2019 were not shown in the table due to limited space

### Linear Mixed Models

In crude models, the number of Ryan White centers per 100,000 population in a county was negatively associated with three-year moving average percentages of LPWA (Crude beta = -0.005, 95% CI: -0.01 ~ 0). In multivariable analysis, five factors were kept in the final model after forward selection, including Hispanic %, Same-sex unmarried partner %, Poverty %, Less than high school education %, and the number of Ryan White centers per 100,000 population. The percentage of less than high school education (adjusted beta = 0.008, 95% CI: 0 ~ 0.015) and the number of Ryan White centers (adjusted beta = -0.006, 95% CI: -0.011~-0.001) were significantly associated with three-year moving average percentages of LPWA. (Table 2)

**Table 2:** The association between county-level characteristics with percentage of LPWA: Forward-based linear mixed effects model

	Crude beta (95% CI)	*p*-value	Adjusted beta (95%CI)	*p*-value
Year	-0.01 (-0.025, 0.006)	0.215	-	-
**Racial residential segregation**			-	-
Black %	-0.002 (-0.004, 0)	0.052	-	-
Hispanic %	0.011 (0, 0.022)	0.057	0.01 (-0.002, 0.021)	0.092
Isolation index			-	-
Black/White dissimilarity index	0.002 (-0.001, 0.006)	0.191	-	-
White/Non-White dissimilarity index	0.003 (-0.001, 0.006)	0.16	-	-
**Social & community context**				
Population density	0 (0, 0)	0.5	-	-
Same-sex unmarried partner %	0.163 (-0.003, 0.33)	0.057	0.12 (-0.052, 0.292)	0.174
Mobile homes %	0 (-0.003, 0.004)	0.84	-	-
**Economic stability**				
Poverty %	-0.002 (-0.007, 0.003)	0.488	0 (0, 0.001)	0.2
Unemployed %	-0.003 (-0.012, 0.007)	0.573	-	-
Median income	0 (0, 0)	0.575	-	-
Gini index	-0.868 (-1.984, 0.248)	0.13	-	-
**Education access**				
Less than high school %	0.003 (-0.004, 0.01)	0.384	0.008 (0, 0.015)	0.046
**Health care access & quality**				
Mental health care providers per 100,000	0.001 (0, 0.001)	0.122	-	-
Shortage of primary care physicians	0.017 (-0.045, 0.08)	0.588	-	-
Shortage of mental healthcare providers	0.002 (-0.049, 0.053)	0.933	-	-
Uninsured %	0.006 (-0.004, 0.016)	0.253	-	-
Ryan White centers per 100,000 population	-0.005 (-0.01, 0)	0.033	-0.006 (-0.011, -0.001)	0.028

Notes: All county-level characteristics were drawn from publicly available datasets for the years 2016–2019

Regarding three-year moving average delay time, counties with a larger isolation index for the Black population (crude beta = 4.947, 95% CI: 0.094 ~ 0.422), larger percentage of unemployment (crude beta = 0.258, 95% CI: 0.094 ~ 0.422), and larger percentage of uninsured (crude beta = 0.308, 95% CI: 0.141 ~ 0.475) were more likely to have longer average delay time based on crude models. In the multivariable model, the significant association of isolation index (adjusted beta = 5.079, 95% CI: 0.268 ~ 9.889) and percentage of uninsured (adjusted beta = 0.273, 95% CI: 0.106 ~ 0.44) with average delay time remained. (Table 3)

**Table 3:** The association between county-level characteristics with delay time (years): Forward-based linear mixed effects model

	Crude beta (95% CI)	*p*-value	Adjusted beta (95%CI)	*p*-value
Year	-0.24 (-0.482, 0.002)	0.054	-	-
**Racial residential segregation**				
Black %	0.037 (-0.003, 0.078)	0.075	-	-
Hispanic %	0.141 (-0.091, 0.373)	0.235	0.21 (-0.027, 0.447)	0.086
Isolation index	4.947 (0.356, 9.538)	0.037	5.079 (0.268, 9.889)	0.041
Black/White dissimilarity index	-0.035 (-0.099, 0.03)	0.297	-	-
White/Non-White dissimilarity index	-0.028 (-0.098, 0.042)	0.432	-	-
**Social & community context**				
Population density	-0.003 (-0.008, 0.002)	0.206	-	-
Same-sex unmarried partner %	1.931 (-1.044, 4.905)	0.206	-	-
Mobile homes %	0.057 (-0.015, 0.129)	0.125	-	-
**Economic stability**				
Poverty %	0.069 (-0.023, 0.161)	0.143	-	-
Unemployed %	0.258 (0.094, 0.422)	0.003	-	-
Median income	0 (0, 0)	0.159	-	-
Gini index	2.02 (-17.858, 21.898)	0.842	-	-
**Education access**				
Less than high school %	0.103 (-0.028, 0.234)	0.125	-	-
**Health care access & quality**				
Mental health care providers per 100,000	-0.006 (-0.017, 0.006)	0.327	-	-
Shortage of primary care physicians	-0.251 (-1.348, 0.845)	0.654	-	-
Shortage of mental healthcare providers	-0.076 (-0.947, 0.795)	0.864	-	-
Uninsured %	0.308 (0.141, 0.475)	0	0.273 (0.106, 0.44)	0.002
Ryan White centers per 100,000 population	0.029 (-0.08, 0.138)	0.603	-	-

Notes: All county-level characteristics were drawn from publicly available datasets for the years 2016–2019

## Discussion

This is one of the first studies to examine multiple racial residential segregation indicators and other SDOH in relation to the average percentage of HIV late presentation and the mean delay time from HIV infection to diagnosis at the county level. Our study found that around 30% of new HIV diagnoses were LPWA in SC, and the mean delay time for people with LPWA was approximately 13 years. Counties with higher residential segregation, indicated by the isolation index, were found to have longer average delay time, even after adjusting for the percentage of uninsured and Hispanics. Regarding other SDOH, the number of Ryan White centers and the percentage of less than high school education attainment were associated with the average percentage of LPWA, and the percentage of uninsured was associated with average delay time only.

Our study adds to evidence from other population-based studies evaluating the impact of racial residential segregation on HIV-related outcomes. The historical legacy of discriminatory housing practices (i.e., redlining) due to structural racism affects every aspect of PWH in the US [24]. Buot et al. found that both White-Black dissimilarity and Black isolation index were predictors for HIV incidence among Black individuals [25]. A case-control study of veterans living with HIV/AIDS reported that a high isolation index was associated with a higher risk of HIV infection in all US geographic divisions, even after controlling for racial identity and neighborhood-level socioeconomic deprivation [26]. Different dimensions of residential segregation are correlated, but each of them represents a distinct geographic residential pattern. Our results suggest that the isolation index instead of other residential segregation measurements (i.e., Black racial composition and White-Black dissimilarity index) impacts the average delay time from HIV infection to initial diagnosis. This could be due to heterogeneity in the types of macro-area units in the analysis and varied HIV outcomes explored. It is postulated that segregation is closely related to neighborhood differences in access to medical, economic, political, and environmental resources, which may influence health disparities [27]. However, the mechanisms through which racial residential segregation affects late HIV presentation warrants continued interest and further investigation. Elucidating the impact of segregation on late HIV presentation could have substantial implications for identifying key intervention points and improving health outcomes among PWH.

We found that counties with a higher percentage of the population with less than a high school education were more likely to have higher percentages of LPWA. Improving education attainment is often cited as a structural strategy for HIV transmission prevention. However, previous findings from area-based studies regarding the impact of education attainment on HIV late presentation were mixed. In a zip code tabulation area (ZCTA)-level study, Sheehan and colleagues found increases in late-stage diagnoses were associated with a higher percentage of individuals with less than a high school education among foreign-born Latinos [8]. Some other researchers found the inverse of our result. For example, one national study using state-level aggregate data reported that a 5% increase in the percentage of the population with less than a high school education was related to a 5% decrease in late-stage diagnoses in the US [28]. Varied age groups and geographic units in different studies might help explain the mixed results. Further studies are needed to examine the interplay of education, HIV late diagnosis, and some other demographic and socioeconomic factors.

With respect to healthcare access and quality-related factors, we found that the number of Ryan White centers per 100,000 people in each county and the percentage of the population uninsured were associated with the percentage of LPWA and the average delay time, respectively. Yusuf et al. explored 332 census tracts in the city of Philadelphia, and results showed one standard deviation increase in distance to adjacent testing centers was related to a 1% higher prevalence of HIV late diagnosis [29]. The Health Resources & Services Administration (HRSA)’s Ryan White HIV/AIDS Program (RWHAP) has developed a wide range of HIV testing and care engagement interventions over the decades [30, 31]. For instance, the AIDS Education and Training Centers (AETC) program, one clinician-training arm of the RWHAP, developed the National HIV Curriculum, which described HIV testing in depth, including HIV screening recommendations, HIV diagnostic tests, and the complexities of test interpretation [31]. Additionally, the National Clinician Consultation Center (NCCC) under AETC provides clinician-to-clinician expert advice on HIV testing and care engagement [32]. Continued efforts should be made by RWHAP to improve HIV testing, and extra attention is needed for counties with fewer Ryan White centers per 100,000 population to decrease HIV late presentation.

There are several limitations that need to be acknowledged in the current study. First, the unit of analysis in this study was county, and there might be different findings when aggregating the data to other geographic levels or more granular unit (census tract level), which is known as the modifiable areal unit problem (MAUP) [33]. Second, we cannot draw any causal relationships for our findings and rule out other unexamined county-level factors. Third, small numbers of HIV diagnoses in some counties may result in greater fluctuations in percentages of LPWA and average delay time in these counties. However, 3-year moving average outcomes were used in our analysis, which could help minimize fluctuation in counties with small numbers of diagnoses. Despite these limitations, our study is strengthened by linked statewide population-based HIV diagnosis data and standard publicly available county-level data. We also examined multiple SDOH and two proxies of HIV late presentation, which may help identify counties in most need of interventions to prevent or reduce HIV late-stage diagnosis.

## Conclusion

Reducing LPWA and associated geographic disparities requires multifaceted intervention that addresses residential segregation and multiple dimensions of SDOH. Further research is needed to understand the etiology of late HIV presentation due to residential segregation and other SDOH (i.e., education attainment and healthcare access) to develop interventions to address county-level context to reduce HIV late presentation.

## Electronic Supplementary Material

Below is the link to the electronic supplementary material.

Supplementary Material 1

## Data Availability

The authors are prohibited from making individual-level data available publicly due to provisions in our data use agreements with state agencies/data providers, institutional policy, and ethical requirements. To facilitate research, we make access to such data available via approved data access requests through the data owners. The data is unavailable externally or for re-release due to prohibitions in data use agreements with our state agencies or other data providers. Institutional policies stipulate that all external requests for data access require collaboration with an (author’s affiliation) researcher. For more information or to make a request, please contact (Bankole Olatosi, PhD): Olatosi@mailbox.sc.edu. The underlying analytical codes are available from the authors on request.

## Declarations

This work was supported by the National Institute of Allergy and Infectious Diseases of the National Institutes of Health [grant number R56AI174896-01A1 to XL and R01AI164947-01to BO]. XY’s efforts were supported by the National Institute of Nursing Research of the National Institutes of Health under Award Number R21NR021079. The content is solely the authors’ responsibility and does not necessarily represent the official views of the National Institutes of Health.
