# Supplementary material for: Association of Racial Residential Segregation and Other Social Determinants of Health with HIV Late Presentation
Source: AIDS Behav. 2024 Oct 17;29(2):517–26. doi: 10.1007/s10461-024-04535-4 (PMC11814007; doi:10.1007/s10461-024-04535-4)
Supplement: Supplementary file 1 — Supplementary Material 1 [file 10461_2024_4535_MOESM1_ESM.docx]

| Supplemental table 1 The definition and data source of each county-level factor | | |
| --- | --- | --- |
| **Variable names** | **Definition** | **Data source** |
| **Racial residential segregation** |  |  |
| Black % | Percentage of the population reporting Black/African American race alone | American Community Survey (ACS) |
| Hispanic % | Percentage of population reporting Hispanic ethnicity | ACS |
| Isolation index | The extent to which the Black members are exposed only to another | Self-developed |
| Black/White dissimilarity index | The larger the number, the greater the residential segregation between Black and White residents. | County Health Rankings (CHR) |
| White/Non-White dissimilarity index | The larger the number, the greater the residential segregation between White and non-White residents. | CHR |
| **Social & community context** |  |  |
| Population density | Number of population per square mile | ACS |
| Same-sex unmarried partner % | Percentage of households with same-sex unmarried partner | ACS |
| Mobile homes % | Percentage of housing units that are mobile homes | ACS |
| **Economic stability** |  |  |
| Poverty % | Percentage of population (age 18 and over) for whom poverty status is determines | ACS |
| Unemployed % | Percentage of civilian labor force (age 16 and over) that is unemployed | ACS |
| Median income | Median household income | ACS |
| Gini index | Income inequality | ACS |
| **Education access** |  |  |
| Less than high school % | Percentage of population (age 25 and over) with less than high school education | ACS |
| **Healthcare access & quality** |  |  |
| Mental health care providers per 100,000 | The number of mental health providers per 100,000 population | ACS |
| Shortage of primary care physicians | Health Professional Shortage Area (HPSA) code - shortage of primary care physicians | Area Health Resources file (AHRF) |
| Shortage of mental healthcare providers | HPSA code - shortage of mental healthcare providers | AHRF |
| Uninsured % | Percentage of population without health insurance coverage | ACS |
| Ryan White centers per 100,000 | the number of Ryan White centers within 25 miles radius of each county per 100,000 population | UN Department of Health and Human Services (DHHS) |
